# Defining adult asthma endotypes by clinical features and patterns of volatile organic compounds in exhaled air

**DOI:** 10.1186/s12931-014-0136-8

**Published:** 2014-11-28

**Authors:** Norbert Meyer, Jan W Dallinga, Sarah Janine Nuss, Edwin JC Moonen, Joep JBN van Berkel, Cezmi Akdis, Frederik Jan van Schooten, Günter Menz

**Affiliations:** High Altitude Clinic (Hochgebirgsklinik) Davos, Davos-Wolfgang, Switzerland; Clinic for Rheumatology, Immunology and Allergology, Divison of Allergology, University Hospital of Bern, Bern, Switzerland; Department of Toxicology, Nutrition and Toxicology Research Institute Maastricht (Nutrim), Maastricht University Medical Center, Maastricht, The Netherlands; Swiss Institute of Allergy and Asthma Research (SIAF), University of Zurich, Christine-Kühne Center for Allergy Research and Education (CK-CARE), Davos, Switzerland

**Keywords:** Asthma, Endotypes, Phenotype, Volatile organic compounds, Exhaled air, Cluster

## Abstract

**Background:**

Several classifications of adult asthma patients using cluster analyses based on clinical and demographic information has resulted in clinical phenotypic clusters that do not address molecular mechanisms. Volatile organic compounds (VOC) in exhaled air are released during inflammation in response to oxidative stress as a result of activated leukocytes. VOC profiles in exhaled air could distinguish between asthma patients and healthy subjects. In this study, we aimed to classify new asthma endotypes by combining inflammatory mechanisms investigated by VOC profiles in exhaled air and clinical information of asthma patients.

**Methods:**

Breath samples were analyzed for VOC profiles by gas chromatography–mass spectrometry from asthma patients (n = 195) and healthy controls (n = 40). A total of 945 determined compounds were subjected to discriminant analysis to find those that could discriminate healthy from asthmatic subjects. 2-step cluster analysis based on clinical information and VOCs in exhaled air were used to form asthma endotypes.

**Results:**

We identified 16 VOCs, which could distinguish between healthy and asthma subjects with a sensitivity of 100% and a specificity of 91.1%. Cluster analysis based on VOCs in exhaled air and the clinical parameters FEV1, FEV1 change after 3 weeks of hospitalization, allergic sensitization, Junipers symptoms score and asthma medications resulted in the formation of 7 different asthma endotype clusters. We identified asthma clusters with different VOC profiles but similar clinical characteristics and endotypes with similar VOC profiles, but distinct clinical characteristics.

**Conclusion:**

This study demonstrates that both, clinical presentation of asthma and inflammatory mechanisms in the airways should be considered for classification of asthma subtypes.

**Electronic supplementary material:**

The online version of this article (doi:10.1186/s12931-014-0136-8) contains supplementary material, which is available to authorized users.

## Background

Asthma is a chronic inflammatory disorder of the airways, which causes typical clinical symptoms like wheezing, breathlessness and chest tightness associated with a reversible obstruction of the airways. The heterogeneity of the clinical dimensions of asthma lead to the definition of clinical asthma phenotypes, classified by steroid response, obesity, fixed airway obstruction or trigger factors like allergens, air pollution, cigarette smoke, aspirin and exercise [1]. Using cluster analyses based on clinical and demographic information, adult asthma patients could be classified into 5 different subgroups [2], and children with severe asthma could be divided into 4 phenotypic clusters [3]. It is assumed that the clinical asthma phenotypes are associated with different inflammatory pathways or molecular mechanisms and on the other hand, similar molecular mechanisms may be present in different asthma phenotypes. In this context, the term “asthma endotypes” was introduced. Endotypes describe disease entities, which are functionally defined by distinct clinical features and pathologically by a molecular mechanism and treatment response [4,5]. It is proposed that the classification of patients into endotypes is important for establishing effective preventive measures and determining appropriate individualized treatments [1]. For quantification of molecular mechanisms non-invasive methods, reflecting the local airway inflammation, are important approaches.

Analysis of volatile organic compounds (VOC) in exhaled breath is a useful and non-invasive method for the assessment of airway inflammation [6]. For the collection of exhaled breath, three to four normal exhalations are necessary, which could be easily performed in children or in severe asthma patients with very low expiratory volumes [7]. VOCs are present in ambient air as well as result from metabolic processes in many cells and microorganisms. They are mainly released during inflammation from cells in response to oxidative stress as a result of activated leukocytes, which produce reactive oxygen species (ROS) [8]. ROS degrade cell membranes by lipid peroxidation and convert polyunsaturated fatty acids to VOCs, which are exhaled in the breath. In the human breath hundreds of different VOCs can be detected, which are divided into hydrocarbons, oxygen, or sulfur containing compounds and nitrogen containing substances [9]. The relative composition and concentrations of VOCs may alter in the presence of diseases and characteristic VOC patterns in lung diseases like lung cancer [10], cystic fibrosis [11], chronic obstructive pulmonary disease [12] and asthma [7] have been demonstrated. VOC profiles in exhaled air may distinguish between asthma patients and patients with chronic obstructive pulmonary disease [13]. In asthma patients, VOC patterns have been identified, which could specifically distinguish between allergic and non-allergic asthma [7] suggesting that different asthma phenotypes may be characterized by different VOC profiles in exhaled air. In addition, a recent study demonstrate that VOC profiles in exhaled air could classify clinically relevant disease phenotypes based on sputum inflammatory profile [14] and that VOC profiles might predict steroid responsiveness in mild/moderate asthma patients [15].

In this study, we measured VOCs in exhaled air from asthma patients and used the VOC profiles together with clinical characteristics for the formation of asthma clusters. We wanted to test the hypothesis that asthma clusters with similar clinical characteristics but different VOC patterns in exhaled air or clusters with similar VOC patterns but different clinical characteristics exist.

## Methods

### Study design and asthma patients

Adult patients had a physician diagnosis of asthma according to GINA guidelines (Global Initiative for Asthma, www.ginasthma.org). All patients included in the study were treated for at least 3 weeks at the high altitude clinic Davos-Wolfgang, which is located at 1600 m above sea level in the Swiss Alpine. The clinical and examination parameters were assessed when the patient arrived and after 3 weeks. All patients were recruited during a period of 8 months. The asthma patients were admitted to the high altitude clinic to optimize their disease. Therefore, mild, moderate and severe asthma patients were included. To exclude chronic obstructive pulmonary disease, asthma was defined by a reversibility in forced expiratory volume in 1 s (FEV1) of at least 12% predicted after inhalation of a short acting β2-agonist. Asthma patients with an acute respiratory infection were excluded. To classify these patients as atopic or non-atopic, we evaluated their medical history, and skin prick tests were performed with animal dander, food allergens, pollens, fungi and latex. The NIOX system from Aerocrine was used for FENO (exhaled nitric oxide) measurements, according to the manufacturer’s instructions. Blood eosinophils and eosininophilic cationic protein (ECP) were analysed in the laboratory of the high altitude clinic. The multidisciplinary treatment at high altitude, consisting of personalised treatment plans with physiotherapy and education, aimed to achieve full asthma control with the lowest possible dose of asthma medication. For the assessment of the level of asthma control the six-item Asthma Control Questionnaire (Junipers symptom score) was used [16]. Responses to each item are rated on a six-point scale and subsequently the mean was calculated, which ranged between 0 (totally controlled) and 6 (severely uncontrolled). Informed consent was obtained from all asthma patients. The main characteristics of the asthma patients are shown in Table 1. The study was approved by the local ethical committees of Graubünden and of Zürich. The data was stored in a database and analyzed by SPSS 18 (SPSS Schweiz AG, Zürich, Switzerland) and Graphpad Prism 4 (Graphprism Software, La Jolla, California). Healthy controls living in Davos were selected without any diseases and no allergies in their history during the same period of the study.

**Table 1:** **Characteristics of asthma patients**

**Allergic asthma**	**77.9%**
Age (years)	49.8 ± 15.2
Age of onset (years)	23.5 ± 17.0
Gender (male)	44.6%
Smoker	4.6%
Long-acting β2 agonist	92.8%
Short-acting β2 agonist (puffs per day)	1.8 ± 3.2
Systemic steroids	26.1%
Inhaled steroids	94.9%
Theophylline	21.0%
Junipers symptoms score	2.2 ± 1.2
ATS	30.2%
Serum ECP (μg/l)	22.9 ± 18.6
Blood eosinophils (%)	5.6 ± 3.8
FEV1 (% of predicted normal value)	83.2 ± 23.5

Percentages of male asthma patients, asthma patients who are active smokers, asthma patients fulfilling ATS (American Thoracic Society) criterias for severe asthma, asthma patients with an allergic asthma and asthma patients with theophylline, inhaled long acting β2 agonists, inhaled steroids or systemic steroids as treatment are shown. In addition, year of onset, age, serum ECP (eosinophilic cationic protein), blood eosinophils, FEV1 values are shown. The units are indicated in the brackets.

### Collection of exhaled air

Exhaled air was collected between 07.00 and 07.30 h from all asthma patients and healthy subjects before breakfast. The subjects were asked to exhale their breath in a resistance-free plastic bag (Tedlar bag, SKC Ltd, Dorset, UK). Three to four exhalations were sufficient to fill the 5 l bag. No special provisions for the mode of exhalation, nor special directions concerning the diet of the subjects were given. The samples of both patients and controls were collected in the same room, to prevent the occurrence of a background bias. Therefore, and because of the data analysis methods used (i.e. discriminant analysis), no background samples were collected. Within 1 h of collection, the bag was emptied under pressure over a stainless-steel two-bed sorption tube, filled with carbograph 1TD/Carbopack X (Markes International, Llantrisant, Wales, UK) and stored at room temperature until analysis.

### VOC’s analysis

For the analyses, the desorption tubes were placed inside the thermal desorption unit (Marks Unity desorption unit, Marks International Limited, Llantrisant, Wales, UK) and quickly heated to 270°C in order to release all VOCs and transport the released VOCs onto the GC-capillary. The used desorption unit was highly suitable for repeated, quantitative and reproducible measurements. Ten percent of the sample was injected into the GC, the remaining 90% transported to another adsorption tube for storage and any later re-analysis. Just before the sample enters the GC, the sample is trapped by a cold trap at 5°C in order to focus and concentrate the sample. The VOCs were separated by capillary gas chromatography (column: RTX-5 ms, 30 m × 0.25 mm 5% diphenyl, 95% dimethylsiloxane, film thickness 1 μm, Thermo Electron Trace GC Ultra, Thermo Electron Corporation, Waltham, USA). The temperature of the gas chromatograph was programmed as follows: 40°C during 5 min, then raised with 10°C/min until a final maximum temperature of 270°C. This temperature was maintained for 5 min. Time-of-flight mass spectrometry (TOF-MS) (Thermo Electron Tempus Plus time-of-flight mass spectrometer, Thermo Electron Corporation, Waltham, USA) was used to detect and identify components available in the samples. Electron ionization mode was set at 70 eV and the mass range *m/z* 35*–*350 was measured at a scan rate of 0.2 s. All chromatograms were preprocessed [6] and combined into a final data matrix, which was used for the discriminant analyses.

### Cluster formation and statistical analyses

Variables for cluster analyses were selected on the basis of their considered contribution for the characterization of asthma endotypes. Categorical variables were standardized to 0 or 1 and continuous variables were standardized to a continuous scale between 0 and 1. For cluster analysis of categorical and continuous variables 2-step cluster analysis approach using the program IBM SPSS Statistics 18.0 (IBM Corporation, New York, United States) was performed. To avoid the formation of very small clusters, the maximum cluster number was limited to 8. Mean values of examination results or clinical features were calculated and are shown as mean ± standard deviation (SD). All statistical analyses were performed using SPSS version 18.0 (IBM) and Graph Prism version 4.0 (GraphPad Software Inc, San Diego, CA).

## Results

### Identification of VOCs implicated in the pathogenesis of asthma

**Figure 1:**
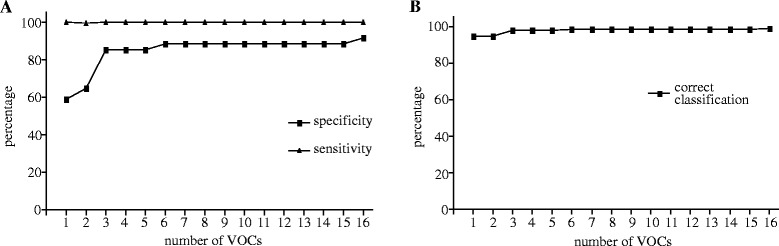
**Course of sensitivity and specificity as a function of the number of VOCs involved in the discriminant analyses.** The sensitivity and specificity to discriminate between asthma and healthy subjects depends on the number of VOCs **(A)**. The correct classification of asthma and healthy subjects as a function of the VOCs is presented **(B)**.

To investigate, which VOCs are implicated during asthmatic airway inflammation, VOCs in exhaled air were compared between asthma patients and healthy controls. A total of 945 VOCs were found. To select the most relevant VOCs, a classification model was constructed based on 16 VOCs. With this model it was possible to distinguish between asthma patients and healthy controls with a sensitivity of 100% and specificity of 91.1% if 16 components were used. 5 components were still sufficient to achieve a sensitivity of 100% and specificity of 85.3% (Figure 1A). This model with 16 VOCs was able to classify healthy and asthma subjects 98.7% correctly. The model with 5 VOCs still classified the subjects 98.0% correctly (Figure 1B). The chemical structures of the VOCs were partly identified and are shown (Additional file 1: Table S1).

### Formation of asthma endotypes

**Figure 2:**
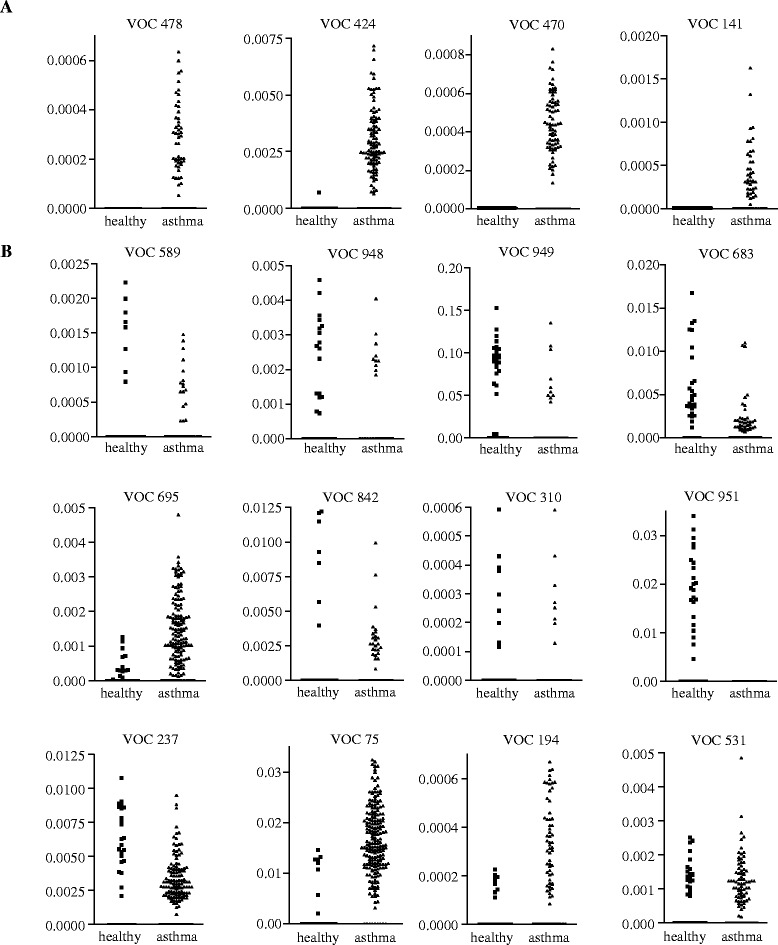
**VOC concentrations in exhaled air in asthma patients and healthy subjects.** VOC in exhaled air were compared between healthy donors and asthma patients. The four VOCs, which have been selected for cluster analyses, are shown in **A**. VOCs not selected for cluster analyses are shown in **B**.

For the formation of asthma endotype clusters by clinical features, asthma medications and VOCs in exhaled air unsupervised hierarchical two-step cluster analysis was used. The clinical features included forced expiratory volume in one-second (FEV1), its ratio to the inspiratory vital capacity (IVC), FEV1 improvement after 3 weeks in the high altitude clinic and Junipers asthma symptom score assessed at admittance. The therapy features included the fraction of patients using systemic steroids, inhaled steroids or long β2 agonists and the frequency of short β2 agonist usage per day. To have an equal proportion between clinical parameters, asthma therapy features and VOCs in exhaled air for cluster analysis, we selected 4 VOCs for cluster analysis, which were detectable in asthma but not in healthy subjects (Figure 2). In detail, VOCs 141, 424, 470, 478 were selected (Figure 2A).

Unsupervised hierarchical two-step cluster analysis of clinical features, asthma medications and 4 VOCs divided the asthma patients into 7 different clusters (Table 2). The two smallest clusters included 14 patients (cluster 4 and 7) and the largest cluster 40 patients (cluster 2). Asthma patients in cluster 6 and 7 have a non-allergic asthma. The asthma patients of cluster 7 have a more severe disease compared to the asthma patients in cluster 6. In detail, asthma patients in cluster 7 have all systemic steroids as medications, a lower FEV1 and higher asthma symptoms scores compared to asthma patients in cluster 6 (Table 2).

**Table 2:** **Values for cluster formation**

**Parameter**	**Cluster 1**	**Cluster 2**	**Cluster 3**	**Cluster 4**	**Cluster 5**	**Cluster 6**	**Cluster 7**
n	33	40	28	14	37	29	14
Allergies	100%	100%	100%	100%	100%	0%	0%
FEV1	99.7 ± 15.8	96.7 ± 16.3	58.7 ± 14.5	105.0 ± 17.2	73.1 ± 18.0	84.4 ± 18.1	58.3 ± 22.5
FEV1/IVC	0.99 ± 0.09	0.98 ± 0.1	0.77 ± 0.15	1.02 ± 0.05	0.91 ± 0.18	0.92 ± 0.17	0.71 ± 0.21
FEV1 improvement	−4.9 ± 11.8	1.3 ± 7.6	8.6 ± 14.6	−0.4 ± 6.9	2.1 ± 14.0	1.0 ± 11.3	14.8 ± 21.3
Junipere	1.8 ± 1.3	1.6 ± 1.0	3.2 ± 0.8	0.9 ± 0.6	2.8 ± 1.0	1.7 ± 0.9	2.9 ± 1.1
Systemic steroids	0%	0%	0%	7.1%	97.3%	0%	100%
Inhaled steroids	100%	100%	100%	42.9%	100%	96.6%	92.9%
Long β2 mimetics	100%	100%	100%	0%	100%	100%	100%
Short β2 mimetics	0.52 ± 0.94	0.68 ± 1.25	3.93 ± 4.74	0.57 ± 1.40	3.05 ± 3.54	1.10 ± 1.76	3.79 ± 5.32
VOC_141	0.015 ± 0.069	0.191 ± 0.246	0.042 ± 0.119	0.020 ± 0.076	0.163 ± 0.381	0.056 ± 0.165	0.034 ± 0.112
VOC_470	0.033 ± 0.090	0.360 ± 0.253	0.238 ± 0.241	0.126 ± 0.228	0.062 ± 0.143	0.214 ± 0.268	0.174 ± 0.251
VOC_478	0	0.152 ± 0.175	0.041 ± 0.112	0.012 ± 0.046	0.053 ± 0.128	0.104 ± 0.179	0.147 ± 0.195
VOC_424	0.436 ± 0.988	3.228 ± 1.751	1.070 ± 1.654	1.938 ± 1.717	0.933 ± 1.502	1.921 ± 2.031	1.816 ± 1.828

Percentages of patients with allergies, systemic steroids, inhaled steroids and inhaled long acting β2 agonists for asthma treatment are shown. FEV1 (forced expiratory volume in 1 second) values are shown in the percentage of the predicted normal values for FEV1. For the Junipers symptoms score and short β2 agonists the mean values ± SD are indicated. Unit for inhalation of short β2 agonists is puffs per day.

In addition, 5 subgroups of patients with an allergic asthma were identified (cluster 1–5). Asthma patients in cluster 3 showed the most severe disease of allergic asthma with a high Junipers symptoms score, a low FEV1 but patients in cluster 3 did not use systemic steroids for asthma therapy. Asthma patients in clusters 1 and 2 had similar clinical features, including good FEV1 values, low Junipers symptoms scores, and no systemic steroid usage as asthma medications. Asthma patients in cluster 4 have a mild allergic asthma with similar clinical characteristics like asthma patients in cluster 1 and 2 but receive different asthma medications. Cluster 5 identifies asthma patients with a moderate allergic asthma having systemic steroids as asthma medication (for more details see Table 2).

More clinical and demographic parameters including age of onset, gender, body mass index (BMI), smoking habits, American thorax society (ATS) criteria for severe asthma, exhaled nitric oxide, serum eosinophilic cationic protein (ECP), circulating eosinophils, IgE serum levels, and usage of theophylline, which were not used for the cluster analysis, are shown in Table 3.

**Table 3:** **Additional clinical characteristics of asthma clusters**

**Parameter**	**Cluster 1**	**Cluster 2**	**Cluster 3**	**Cluster 4**	**Cluster 5**	**Cluster 6**	**Cluster 7**	**n**
Age of onset (years)	20.7 ± 16.3	19.5 ± 15.4	19.0 ± 17.9	26.5 ± 20.5	24.1 ± 17.1	28.9 ± 14.8	34.3 ± 16.2	195
Gender (male)	36.4%	52.5%	60.1%	35.7%	37.8%	51.7%	21.4%	195
Age	41.6 ± 15.9	46.4 ± 15.9	56.6 ± 12.1	47.3 ± 14.6	49.5 ± 13.4	54.4 ± 12.4	58.9 ± 16.4	195
BMI	25.0 ± 3.6	25.5 ± 3.1	26.0 ± 4.3	25.9 ± 4.2	25.3 ± 4.7	24.7 ± 3.8	24.7 ± 5.0	195
Tobacco exposure (py)	4.2 ± 6.0	3.3 ± 7.7	6.0 ± 8.0	1.8 ± 4.1	4.2 ± 7.1	5.0 ± 8.6	2.0 ± 3.8	195
Smoker	6.1%	2.5%	7.1%	7.1%	5.4%	3.5%	0%	195
ATS	9.1%	12.5%	35.7%	0%	75.7%	6.9%	78.6%	195
Exhaled NO (ppb)	36 ± 29	32 ± 18	34 ± 30	27 ± 27	43 ± 36	28 ± 18	41 ± 26	195
ECP	27.0 ± 27.0	21.7 ± 13.8	26.2 ± 20.8	11.9 ± 7.2	22.3 ± 16.1	18.9 ± 15.6	31.1 ± 19.8	194
Eosinophils	6.5 ± 4.5	5.6 ± 3.1	6.3 ± 3.6	4.8 ± 5.0	4.8 ± 3.1	4.8 ± 3.8	7.2 ± 4.2	195
IgE	1383 ± 4731	524 ± 883	648 ± 1007	304 ± 340	392 ± 877	80 ± 103	140 ± 262	194
Theophyllin	12.1%	12.5%	32.1%	0%	32.4%	10.3%	57.1%	195

Percentages of male asthma patients, asthma patients who are active smokers, asthma patients fulfilling ATS (American Thoracic Society) criterias for severe asthma and asthma patients with theophylline treatment are shown. In addition, the BMI (body mass index), year of onset, age, exhaled NO (nitric oxide), serum ECP (eosinophilic cationic protein), blood eosinophils, IgE serum levels are shown as mean values ± SD. The units are indicated in the brackets. Lifetime tobacco exposure is indicated in pack years (py).

### Characterization of VOC profiles in asthma endotypes

**Figure 3:**
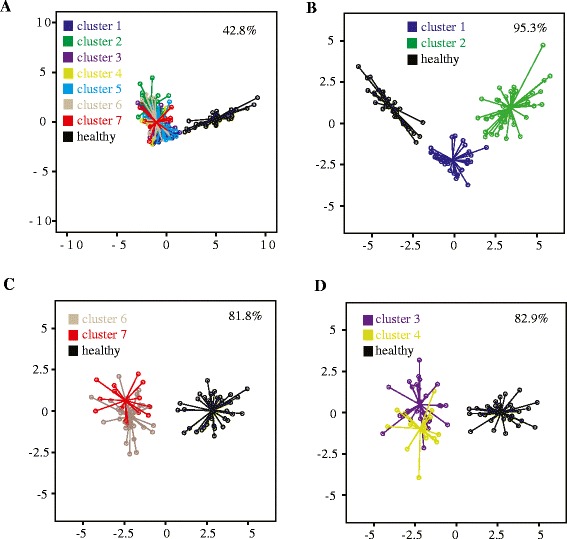
**Discriminant analyses for asthma clusters.** Discrimination analyses between all asthma clusters and healthy patients **(A)** and between indicated asthma subgroups **(B, C, D)** are shown. The percentage indicates the correct classification. Clusters shown in **B** have similar phenotype characteristics whereas clusters shown in **C** and **D** have different phenotype characteristics (see Table 2).

The concentrations of VOCs in the asthma clusters are shown in Table 2. To investigate if the VOCs were different or similar in the identified asthma clusters, we analysed if the VOCs could discriminate between them. Linear discriminant analysis demonstrates that 42.8% of all asthma patient clusters and healthy subjects could be correctly classified by VOCs in the exhaled air (Figure 3A). However, the correct classification was different between the clusters indicating that there exist clusters with similar VOC profiles and clusters with different VOC profiles. Subsequent analyses demonstrate that cluster 1 and 2 have similar clinical features (Table 2) but different VOC patterns in exhaled air (Figure 3B). Profiles of VOCs can discriminate between cluster 1, 2 and healthy subjects with an high accuracy of 95.3% (Figure 3B). In contrast, the correct classification of asthma patients belonging to cluster 6 and 7 and healthy donors was only 81.8% and there was much overlap of the VOC patterns between these clusters indicating that these asthma clusters have similar VOC patterns in exhaled air (Figure 3C). Interestingly, asthma patients belonging to cluster 6 and 7 have distinct clinical and treatment characteristics (see Table 2). Similar results were found for cluster 3 and 4 (Figure 3D), which are clinical different but have a quite related profile of VOCs in exhaled air as indicated by the relatively low accuracy of 82.9% to distinguish between the asthma patients clusters and healthy donors.

In addition, a second possibility of cluster analysis was performed. In this data set, the following additional parameters were included: age of onset, blood eosinophils, body mass index (BMI), serum IgE levels, exhaled nitric oxide (FeNO) and asthma control. Because unsupervised cluster resulted in the formation of only 3 clusters, a 2-step cluster analysis of this data set using a user-defined cluster number of 7 was performed. We have detected two clusters with a non-allergic asthma and 5 clusters with an allergic asthma (see Additional file 1: Table S2). Again, clusters with a high clinical similarity but distinct profiles of exhaled VOCs (e.g. cluster 2 and 3, Additional file 1: Figure S1B) and clusters with similar VOCs patterns in exhaled air but distinct clinical and treatment characteristics (e.g. clusters 2 and 4, Additional file 1: Figure S1C) are present. Interestingly, cluster 2 and 3 have not only distinct VOCs profiles, they also differ in total IgE levels and blood eosinophils. In contrast, patients in cluster 2 and 4 are clinical different and have similar VOC profiles and IgE/blood eosinophil levels.

## Discussion

The current study identifies VOCs in exhaled air, which could distinguish between asthma patients and healthy subjects with a sensitivity of 100% and specificity of 91%. The combination of clinical asthma features, asthma therapy and exhaled VOCs classified asthma patients into 7 endotype clusters. We identified asthma clusters with different VOC profiles but similar clinical characteristics and clusters with similar VOC profiles, but distinct clinical characteristics.

The activation of different cellular and molecular pathways by different environmental factors suggests that asthma phenotypes are associated with certain inflammatory pathways in the lungs. Asthma endotypes classify asthma patients according to clinical features, treatment response and underlying molecular mechanisms and therefore address molecular mechanism [4]. Accordingly, we used 13 parameters of clinical features, asthma treatment features and VOCs in exhaled air for cluster analysis and classified all asthma patients into 7 different clusters. Recent phenotype studies of severe adult asthma patients or asthmatic children identified 4–5 asthma groups by cluster analysis without addressing molecular mechanisms or including exhaled VOCs for cluster analyses [2,3]. The identification of asthma patient clusters with similar clinical and treatment characteristics but different VOC profiles in the exhaled air indicates that asthma phenotypes might be further divided into subgroups by their exhaled VOC profiles. This finding supports the hypothesis that one asthma phenotype might be characterized by several inflammatory mechanisms [5]. The identification of molecular mechanisms specific for asthma endotypes and diagnostic biomarkers that can discriminate between distinct disease endotypes will be necessary for the development of new asthma therapies [17]. We also detected asthma clusters with similar VOC patterns in the exhaled air but different clinical features suggesting that these VOCs profiles may be specific for airway inflammation, which is found in many asthma phenotypes.

In this study VOCs in exhaled air were analysed for the assessment of airway inflammation because VOCs mostly derive from biological processes such as lipid peroxidation. Pathologic processes have the potential to influence VOCs either by producing new volatile substances or by the metabolic consumption of VOC substrates that are normally present. Volatile products formed during lipid peroxidation include ethane, pentane, hexanal, octanal, nonanal, propanol and butanol [18,19]. Already in the early 80s, pentane and ethane levels in exhaled air were described as markers for *in vivo* peroxidation [8]. Ethane was described to be elevated in the breath of patients with chronic obstructive pulmonary disease (COPD) and asthma [20,21]. However, measuring a single compound in exhaled air might not be sufficient to monitor complex and heterogeneous diseases. In addition, some VOCs in exhaled air are not only increased during airway inflammation. For example, pentane appears to be relatively non-specific VOC for airway inflammation because its levels are also raised in other inflammatory conditions such as inflammatory bowel disease [22] or rheumatoid arthritis [23]. Exploring the total amount of VOCs is expected to generate more adequate information about the complexity of processes underlying the pathophysiology of respiratory diseases [24]. In our study the most important 16 VOCs could distinguish with a high sensitivity and specificity between healthy and asthmatic subjects indicating that the VOCs are useful for non-invasive exploration of various biochemical pathways activated in asthma.

Certain inflammatory processes may reflect the involvement of different inflammatory sputum cells. Thus, it has been demonstrated that VOC profiles in exhaled air could classify clinically relevant disease phenotypes based on sputum inflammatory profiles by predicting both neutrophilic and eosinophilic sputum cell phenotypes of asthma patients [14]. However, a variety of interactions among distinct populations of stromal cells, epithelial cells and leukocytes contribute to inflammation in the asthmatic lung [25] and may not be assessed by characterization of cells derived from sputa. Analysis of gene expression in asthmatic biopsies might be a useful technique to understand the inflammatory processes implicated in airway inflammation [26,27]. However, obtaining lung biopsies is invasive and cannot be assessed in all asthma patients. In contrast, breath analysis could detect VOC profiles in a non-invasive and straightforward way.

Although this study does not identify VOCs, which indicate specific inflammatory mechanisms in the airways, we demonstrate that VOCs patterns are an attractive approach for the classification of asthma patients as they might reflect inflammatory processes in the airways and could be collected easily in a non-invasive way. If we include biological relevant features of blood analyses like blood eosinophils and IgE serum levels into the cluster analyses, we detect clusters having similar clinical asthma features (see Additional file 1: Table S2) but differ in IgE levels, blood eosinophils and VOCs suggesting that certain VOCs profiles might indicate IgE- and eosinophil-mediated inflammation. The identification of VOCs as markers for certain inflammatory pathways in asthma patients (e.g. Th2- or Th-17 cell mediated inflammation) might be the basis to guide the patients individual therapy.

Therefore the identification of VOCs, which are specifically related to the expression of certain genes and proteins is one important step in the future, which could be helpful for the identification of discrete pathogenic pathways specific for asthma endotypes.

## Additional file

Additional file 1: Table S1. Identified chemical VOC structures. **Table S2.** Second possibility of cluster analysis. **Figure S1.** Discriminant analyses for asthma clusters.
