# Supplementary material for: Defining adult asthma endotypes by clinical features and patterns of volatile organic compounds in exhaled air
Source: Respir Res. 2014 Nov 28;15(1):136. doi: 10.1186/s12931-014-0136-8 (PMC4264530; doi:10.1186/s12931-014-0136-8)
Supplement: Additional file 1: Table S1. — Identified chemical VOC structures. Table S2. Second possibility of cluster analysis. Figure S1. Discriminant analyses for asthma clusters. [file 12931_2014_136_MOESM1_ESM.docx]

| VOC number | Chemical structure |
| --- | --- |
| 75 | Unknown |
| 683 | Dodecane |
| 949 | Phenol |
| 951 | Quinoline, decahydro-, |
| 141 | 1-Dodecanol, 3,7,11-trimethyl- |
| 470 | Unknown |
| 948 | 5-Hexenoic acid |
| 695 | Benzene |
| 589 | 2-Propionyloxypentadecane |
| 478 | Unknown |
| 842 | Tetradecanoic acid |
| 310 | 2-Butyl-2,7-octadien-1-ol |
| 237 | Octanal |
| 424 | 1,3-Dioxolane, 2-(phenylmethyl)- |
| 194 | 4-Cyclopentene-1,3-dione, 4-phenyl- |
| 531 | Heptane, 2,4-dimethyl- |

Table S1: *Identified chemical VOC structures.*

| Parameter | Cluster 1 | Cluster 2 | Cluster 3 | Cluster 4 | Cluster 5 | Cluster 6 | Cluster 7 |
| --- | --- | --- | --- | --- | --- | --- | --- |
| n | 14 | 29 | 33 | 39 | 37 | 18 | 24 |
| Allergies | 100% | 100% | 100% | 100% | 97.3% | 0% | 0% |
| FEV1 (% predicted) | 105.0±17.2 | 99.1±18.3 | 100.9±14.4 | 67.7±18.3 | 72.0±18.0 | 96.4±11.1 | 61.5±17.4 |
| FEV1/IVC (% predicted) | 1.02±0.05 | 0.99±0.10 | 0.99±0.09 | 0.83±0.16 | 0.91±0.18 | 1.03±0.07 | 0.71±0.16 |
| FEV1 improvement (%) | -0.4±6.9 | -6.3±12.2 | -0.2±6.4 | 8.1±12.8 | 3.0±14.7 | -1.9±8.3 | 10.0±18.7 |
| Junipere | 0.93±0.62 | 1.9±1.4 | 1.2±0.8 | 3±0.86 | 2.9±1.0 | 1.3±1.0 | 2.6±0.8 |
| Systemic steroids (%) | 7.1% | 0% | 3% | 0% | 97.3% | 5.6% | 50% |
| Inhaled steroids (%) | 48.9% | 100% | 100% | 100% | 97.3% | 94.4% | 100% |
| Long β2 mimetics (%) | 100% | 100% | 100% | 100% | 100% | 100% | 100% |
| Short β2 mimetics (puffs per day) | 0.57±1.4 | 0.4±0.9 | 0.4±1.0 | 3.3±4.2 | 3.6±4.7 | 0.8±1.6 | 2.1±2.4 |
| VOC_141 | 0.020  ±  0.076 | 0.018  ±  0.074 | 0.121  ±  0.208 | 0.117  ±  0.217 | 0.163  ±  0.381 | 0.078  ±  0.202 | 0.029  ±  0.095 |
| VOC_478 | 0.012  ±  0.046 | 0 | 0.148  ±  0.189 | 0.063  ±  0.113 | 0.050  ±  0.128 | 0.052  ±  0.112 | 0.172  ±  0.212 |
| VOC_470 | 0.126  ±  0.228 | 0.037  ±  0.096 | 0.343  ±  0.251 | 0.259  ±  0.255 | 0.052  ±  0.133 | 0.183  ±  0.269 | 0.225  ±  0.260 |
| VOC_424 | 1.938  ±  1.717 | 0.120  ±  0.454 | 3.766  ±  1.403 | 1.248  ±  1.544 | 0.916  ±  1.482 | 2.332  ±  2.350 | 1.536  ±  1.594 |
| Age of onset (years) | 26.5±20.5 | 17.6±14.2 | 18.0±15.3 | 23.0±18.1 | 24.5±17.0 | 30.8±14.0 | 30.8±16.8 |
| Blood eosinophils (%) | 4.8±5.0 | 7.4±4.9 | 5.3±3.3 | 5.7±2.8 | 4.9±3.1 | 4.1±3.0 | 6.8±4.4 |
| BMI (kg/m2) | 25.9±4.2 | 24.8±3.8 | 25.2±3.0 | 26.2±3.9 | 25.7±5.2 | 25.2±4.4 | 23.8±2.7 |
| IgE (kU/l) | 304±340 | 1669±5044 | 415±535 | 566±1002 | 393±877 | 118±245 | 87±92 |
| Exhaled NO (ppb) | 27±27 | 35±28 | 29±17 | 37±29 | 44.8±35.4 | 21±9 | 39±25 |
| Asthma control | UC: 0%  PC: 21.4%  FC: 78.6% | UC: 20.7%  PC: 48.3%  FC: 31.0% | UC: 12.1%  PC: 48.5%  FC: 39.4% | UC: 79.5%  PC: 20.5%  FC: 0% | UC: 75.7%  PC: 21.6%  FC: 2.6% | UC: 0%  PC: 72.2%  FC: 27.8% | UC: 62.5%  PC: 37.5%  FC: 0% |

Table S2: *Second possibility of cluster analysis.*

Percentages of patients with allergies, systemic steroids, inhaled steroids and inhaled long acting β2 agonists for asthma treatment are shown. FEV1 (forced expiratory volume in 1 second) values are shown in the percentage of the predicted normal values for FEV1. For the Junipers symptoms score and short β2 agonists the mean values ± SD are indicated. Unit for inhalation of short β2 agonists is puffs per day. BMI: body mass index. Units for exhaled NO, blood eosinophils, age of onset and BMI are indicated in the brackets. Asthma control (according to GINA guidelines): UC: uncontrolled; PC: partially controlled; FC: fully controlled

Supplementary figure legend:

*Figure S1: Discriminant analyses for asthma clusters.*

Discrimination analyses between all asthma clusters and healthy patients (A) and between indicated asthma subgroups (B, C) are shown. The percentage indicates the correct classification. Clusters shown in B have similar phenotype characteristics whereas clusters shown in C have different phenotype characteristics (see supplementary table S2).
